# Linguistic Variables and Gender Differences Within a Messenger-Based Psychosocial Chat Counseling Service for Children and Adolescents: Cross-Sectional Study

**DOI:** 10.2196/51795

**Published:** 2024-01-12

**Authors:** Zeki Efe, Sabrina Baldofski, Elisabeth Kohls, Melanie Eckert, Shadi Saee, Julia Thomas, Richard Wundrack, Christine Rummel-Kluge

**Affiliations:** 1 Department of Psychiatry and Psychotherapy Medical Faculty Leipzig University Leipzig Germany; 2 Department of Psychiatry and Psychotherapy University Leipzig Medical Center Leipzig University Leipzig Germany; 3 Krisenchat gGmbH Berlin Germany; 4 Department of Psychology Chair of Personality Psychology Humboldt Universität zu Berlin Berlin Germany

**Keywords:** e-mental health, chat counseling, crisis, helpline, linguistic, language, Linguistic Inquiry and Word Count, LIWC, psychiatric symptoms

## Abstract

**Background:**

Text messaging is widely used by young people for communicating and seeking mental health support through chat-based helplines. However, written communication lacks nonverbal cues, and language usage is an important source of information about a person’s mental health state and is known to be a marker for psychopathology.

**Objective:**

The aim of the study was to investigate language usage, and its gender differences and associations with the presence of psychiatric symptoms within a chat counseling service for adolescents and young adults.

**Methods:**

For this study, the anonymized chat content of a German messenger–based psychosocial chat counseling service for children and adolescents (“*krisenchat*”) between May 2020 and July 2021 was analyzed. In total, 661,131 messages from 6962 users were evaluated using *Linguistic Inquiry and Word Count*, considering the following linguistic variables: first-person singular and plural pronouns, negations, positive and negative emotion words, insight words, and causation words. Descriptive analyses were performed, and gender differences of those variables were evaluated. Finally, a binary logistic regression analysis examined the predictive value of linguistic variables on the presence of psychiatric symptoms.

**Results:**

Across all analyzed chats, first-person singular pronouns were used most frequently (965,542/8,328,309, 11.6%), followed by positive emotion words (408,087/8,328,309, 4.9%), insight words (341,460/8,328,309, 4.1%), negations (316,475/8,328,309, 3.8%), negative emotion words (266,505/8,328,309, 3.2%), causation words (241,520/8,328,309, 2.9%), and first-person plural pronouns (499,698/8,328,309, 0.6%). Female users and users identifying as diverse used significantly more first-person singular pronouns and insight words than male users (both *P*<.001). Negations were significantly more used by female users than male users or users identifying as diverse (*P*=.007). Similar findings were noted for negative emotion words (*P*=.01). The regression model of predicting psychiatric symptoms by linguistic variables was significant and indicated that increased use of first-person singular pronouns (odds ratio [OR] 1.05), negations (OR 1.11), and negative emotion words (OR 1.15) was positively associated with the presence of psychiatric symptoms, whereas increased use of first-person plural pronouns (OR 0.39) and causation words (OR 0.90) was negatively associated with the presence of psychiatric symptoms. Suicidality, self-harm, and depression showed the most significant correlations with linguistic variables.

**Conclusions:**

This study highlights the importance of examining linguistic features in chat counseling contexts. By integrating psycholinguistic findings into counseling practice, counselors may better understand users’ psychological processes and provide more targeted support. For instance, certain linguistic features, such as high use of first-person singular pronouns, negations, or negative emotion words, may indicate the presence of psychiatric symptoms, particularly among female users and users identifying as diverse. Further research is needed to provide an in-depth look into language processes within chat counseling services.

## Introduction

Childhood and adolescence are known for their biological, social, and psychological changes as vulnerable periods, in which young people are at an increased risk for experiencing mental health problems. It is also known that an early age of onset of mental illness is a risk factor for poor mental health conditions in adulthood [1,2]. The use of mental health care services for adolescents and young adults can have a positive influence on their attitudes, beliefs, and behaviors, which are known to be important predictors of their later mental health [3]. A growing number of studies indicate that children, adolescents, and young adults use the internet to seek help for their mental health problems because the digital environment is familiar and easily accessible, offers anonymity, and accommodates their need for independence [4-8]. Nearly all young people aged 12 to 19 years in Germany (94%) own a smartphone [9].

With the increased use of smartphones, text messaging has become the primary communication tool for today’s youth [10]. Studies on text messaging usage with mobile phones have shown that adolescents experience text messaging as a quick, easy, convenient, playful, and inexpensive way of communication [11-14]. In line with this trend, a number of crisis helplines and similar services have begun to offer online support services such as chat or email counseling [15-18]. Studies have shown that adolescents prefer texting to talking when seeking help for mental health problems and find it easier to write than to express serious concerns verbally [18,19]. Recent studies support the acceptance, feasibility, and usability of online support services, especially among young people [7,20].

However, written language lacks nonverbal stimuli. Recent research has shown that facial expressions and prosody have an influence on the recognition of a speaker’s intention in face-to-face communication [21,22]. In fact, in comparison to face-to-face interactions, people report higher levels of miscommunication when texting. This might represent a barrier in messenger-based counseling and may make it difficult for young people to understand and interpret the intentions of online counselors [23,24]. This lack of nonverbal cues can also make it more difficult for crisis line counselors to establish and maintain a therapeutic relationship [25,26]. In some studies, counselors reported greater difficulty and a lower perceived ability to establish a therapeutic relationship in the digital environment compared to a face-to-face counseling or therapy setting [25,27]. In addition to nonverbal stimuli, language usage is an important source of information in the therapeutic context, and the way people use words conveys a great deal of information about themselves and their current situation [28]. Language reflects both conscious and unconscious thoughts and feelings [29]. Linguists distinguish 2 aspects in the study of language: the formal and the content features of language. The formal aspect concerns grammar, syntax, reaction speed, speech tempo, etc, whereas content features consider vocabulary and word choice [30]. The investigation of “lexical diversity” thus allows a better insight into the cognitive diversity of people [31,32].

Thus, in the 1970s, it was evidenced for the first time that language use can be a specific marker for psychopathology, especially depression [30]. It was found that individuals with depression use more first-person singular pronouns (ie, “I,” “my,” “me,” and “mine”) in both spoken and written language [33,34], supporting cognitive theories of depression [33], which indicate that depression is associated with an increased self-focus. Recent research has shown that increased use of certain words, for example, sad (eg, “crying,” “grief,” and “sad”) or sleep (eg, “asleep” and “bed”), correlates positively with higher levels of depressive symptoms [35]. Further studies also found gender differences in language usage. For example, it was found that women tend to use more language related to thoughts, emotions, senses, negations, and verbs in the present or past tense than men [36]. Furthermore, women were shown to be more likely to use first-person singular pronouns than men, which is consistent with the higher prevalence of depression in women [36,37]. Besides first-person singular pronouns and negative emotion words, causation words (eg, “because”) were also found to be used more by people having depression [34,38]. In line with this, research confirmed the Seligman theory for *learned helplessness*, which postulates that individuals at risk for depression attribute the cause of a negative event as being internal, global, and stable, by showing that young adults with negative attributional styles were more likely to develop clinically significant depression than those without such attributional styles [39]. There are also studies indicating negative attributional styles as predictive factors for developing depressive symptoms when experiencing negative life events [40-42]. Due to the trend of digitalization in mental health care, linguistic investigations have been conducted in the digital environment as well. In doing so, a positive association was found between Twitter posts indicating loneliness and mental health problems of the users [43]. In another study, which examined the language usage of users of loneliness forums, it was found that these users tended to use words associated with sadness or a desire for social contact, that is, their overall language leaned toward words with negative valence [44]. Regarding associations with psychopathology, the newest findings indicate that individuals with depressive symptoms used fewer complex syntactic constructions, such as adverbial phrases, perhaps because these require greater cognitive effort [35]. The population of young smartphone users and texters remains a vulnerable and underserved group in crisis counseling, which is why further research on outcomes and the effectiveness of specific communication and counseling strategies is needed [45,46]. To date, there have been no attempts to examine the chat content of crisis counseling services with regard to their linguistic structure. For this purpose, anonymized chat messages from a messenger-based psychosocial chat counseling service, *krisenchat* (German for “crisis chat”), were used to examine (1) which linguistic indicators and gender differences can be identified within the messages of chat users and (2) how these linguistic indicators are associated with the presence of psychiatric symptoms. Based on the existing literature, it was hypothesized that female users would be more likely to use first-person singular pronouns, negations, and insight words than male users and users identifying as diverse. Additionally, it was hypothesized that higher use of first-person singular pronouns, negations, negative emotion words, and words indicating causation would be associated with a higher likelihood of the presence of psychiatric symptoms among users.

## Methods

### Sampling and Data Collection

For the purpose of this study, anonymized chat data from all users receiving counseling between May 2020 and July 2021 were extracted from the *krisenchat* database. Data extraction and preparation were performed by authors affiliated with *krisenchat* (ME, SS, JT, and RW) so that chat content remained within the *krisenchat* database. The anonymized chat data included metadata on the chat (total number of messages and words sent by users during the whole counseling process, and number of sessions) and information about the user that counselors identified and noted during the counseling process (sociodemographic information, such as gender and age, and topics of users’ concerns). *krisenchat* counselors were volunteers and had a background in psychosocial studies. In addition, they underwent a structured 2-month training in chat-based counseling. Regarding gender, counselors had 3 options (male, female, and diverse) to mark in their documentation. They were encouraged to record the identified gender and not the biological sex of the users. The gender “diverse” included individuals identifying as nonbinary or diverse, or indicating to be unsure about their gender identity. For more information about the study design and the nature of *krisenchat*, we referred to the initial evaluation study of *krisenchat* [20]. Linguistic variables were determined using *Linguistic Inquiry and Word Count* (LIWC; see below for details).

The sample examined in this study was based on the previous evaluation of *krisenchat*, in which the sample consisted of those who completed a subsequent feedback survey after the counseling session [20]. Thus, out of a total of 11,031 users in the above-mentioned time period, 6962 (63.1%) completed a feedback survey. The chat messages of these 6962 users were analyzed. In total, 661,131 messages (mean 94.96, SD 259.46) from 26,614 chat sessions (mean 3.82, SD 6.24) with a total word count of 8,872,154 (mean 1274.37, SD 2954.57) were analyzed.

### Ethical Considerations

The Medical Faculty of the University of Leipzig approved this study on August 3, 2021 (372/21-ek). Users were informed about the data protection and privacy policy of *krisenchat* when they first contacted the counseling service. The chat counseling only began after confirming the policy with “Yes.” Participants in the study confirmed informed consent via an opt-in function before taking part in the feedback survey.

### Measures

#### Linguistic Variables

LIWC is a software for dictionary-based quantitative text analysis [29]. LIWC performs an automated 1-word analysis based on a lexicon with more than 80 categories (ie, language variables, descriptors, linguistic dimensions, psychological dimensions, concerns, informal language, and punctuation) including a total of 18,711 words. In 2008, the German version of the lexicon was developed, and good equivalence was confirmed for the majority of LIWC categories [47]. The tool has been used in various studies on personality, social, and clinical psychological frameworks and for the analysis of therapeutic essays, everyday communication, or computer-based communication, and it can therefore be considered a reliable software program for quantitative text analysis [47-52].

LIWC counts the number of words within the lexicon over a whole chat and assigns them to categories. The output file includes all categories of the lexicon. All variables, except summary variables, are expressed as percentages of the total word count of a respective chat. Based on previous findings [34-37,44,53,54], the following linguistic variables were considered in this study: first-person singular (eg, “I,” “me,” and “mine”) and first-person plural pronouns (eg, “we,” “us,” and “our”), negations (eg, “no,” “not,” and “never”), positive emotion words (eg, “love,” “nice,” and “sweet”), negative emotion words (eg, “hurt,” “worried,” and “sad”), cognitive process words such as words related to insight (eg, “think” and “know”), and words related to causation (eg, “because” and “effect”).

#### Psychiatric Symptoms

The presence of psychiatric symptoms was assessed during the counseling process and noted by *krisenchat* counselors. The identification of psychiatric symptoms was derived from the concerns reported by the users. Counselors distinguished between the presence of the following symptoms: depression, anxiety, suicidality, self-harm, addictive behavior, eating disorders, flashbacks, and obsessive-compulsive symptoms. Additionally, symptoms were summed up into a dichotomous variable “psychiatric symptoms” to indicate the presence or absence of psychiatric symptoms (0, “not present;” 1, “present”).

### Statistical Analysis

Statistical analyses were performed using IBM SPSS Statistics version 27.0 (IBM Corp). A 2-tailed α value of .05 was applied to statistical testing. First, descriptive statistics were performed for sociodemographic variables and linguistic variables of the total sample. Additionally, Kruskal-Wallis *H* tests (because of nonnormality of the linguistic variables) were used to identify gender differences in use, that is, metadata for the number of sessions, messages, and words of each user. Then, a 1-way multivariate analysis of variance (1-way MANOVA) was conducted to test for gender differences in language usage controlling for word count. Gender was considered as an independent variable, and all 7 linguistic variables (ie, first-person singular pronouns, first-person plural pronouns, negations, positive emotion words, negative emotion words, insight words, and causation words) were considered as dependent variables. Post-hoc univariate ANOVAs were conducted separately for every linguistic variable. Bonferroni correction was applied to account for multiple testing. Then, binary logistic regression analysis was conducted to examine the predictive effect of linguistic variables (first-person singular and plural pronouns, negations, positive and negative emotion words, insight words, and causation words), age, and gender (recoded into a set of dummy variables with “male” as the reference variable) on the presence of psychiatric symptoms. The amount of explained variance as shown by Nagelkerke R^2^ was interpreted as follows: R^2^ >0.20, “acceptable” or small effect size; R^2^ >0.40, “good” or average effect size; and R^2^ >0.50, “very good” or large effect size [55]. Additionally, Spearman correlation coefficients (*ρ*) were reported between linguistic variables. Finally, with the aim to examine the deeper relationship between linguistic variables and psychiatric symptoms, explorative Spearman correlations (*ρ*) between all 7 linguistic variables and all categories of psychiatric symptoms (suicidality, self-harm, depression, anxiety, eating disorder symptoms, flashbacks, obsessive-compulsive symptoms, and addictive behavior) were computed and interpreted as follows: *ρ*=0.10, small effect size; *ρ*=0.30, moderate effect size; and *ρ*=0.50, large effect size [56].

## Results

### Sociodemographic Characteristics

The average user was 17 years old (mean 16.55, SD 3.45 years; range 8-25 years), and most users were female (female: 4988/5978, 83.4%; male: 881/5978, 14.7%; diverse: 109/5978, 1.8%). A large number of all users (4841/6962, 69.5%) contacted the counseling service due to psychiatric symptoms. Further concerns identified were psychosocial distress (eg, school-related problems, family-related problems, bullying, etc; 2370/6962, 34.0%) or emotional distress (eg, grief, lovesickness, anger, and loneliness; 2101/6962, 30.2%) [20].

The users participated in an average of 3.82 (SD 6.24) counseling sessions and sent an average of 94.96 (SD 259.46; range 2-11,512) messages with an average of 1274.37 (SD 2954.57) words throughout the counseling process. Additional testing indicated that there were gender differences in the numbers of sessions (χ^2^_2_=22.849; *P*<.001), messages (χ^2^_2_=14.863; *P*<.001), and words (χ^2^_2_=33.036; *P*<.001). The results are presented in Table 1. Subsequent post-hoc tests indicated that female users attended a significantly higher number of sessions (*z*=−4.211; *P*<.001; *r*=0.05) and sent significantly more messages (z=−3.247; *P*<.001; *r*=0.04) and words (z=−5.349; *P*<.001; *r*=0.07) than male users, whereas there were no significant differences between female users and users identifying as diverse (number of sessions: *P*=.13; number of messages: *P*=.18; number of words: *P*=.22). Users identifying as diverse also attended a significantly higher number of sessions (z=−3.441; *P*<.001; *r*=0.04) and sent significantly more messages (*z*=−2.972; *P*<.001; *r*=0.04) and words (*z*=−3.639; *P*<.001; *r*=0.05) than male users.

**Table 1:** Gender-specific differences in metadata (N=5978).

Metadata variables	Male^a^, mean (SD)	Female^a^, mean (SD)	Diverse^a^, mean (SD)	χ^2^ (*df*)^b^	*P* value
Session count	3.23 (4.17)^c^	4.17 (6.94)^d^	5.43 (7.56)^d^	22.849 (2)	<.001
Message count	79.05 (145.20)^c^	103.74 (287.43)^d^	189.14 (512.32)^d^	14.863 (2)	<.001
Word count	1068.80 (1965.98)^c^	1392.55 (3279.22)^d^	2166.18 (4653.08)^d^	33.036 (2)	<.001

^a^Reduced sample size owing to missing data on gender.

^b^Test statistic for the Kruskal-Wallis *H* test.

^c,d^Different letters indicate significant differences between the groups, while same letters indicate no significant differences between the groups.

### Descriptive Statistics of Linguistic Variables

In the total sample, the mean percentage of first-person singular pronouns among all words of a user during the whole counseling process was 11.59% (SD 2.46%), indicating that on average, more than one-tenth of all words written throughout all chat messages was a first-person singular pronoun (“I,” “me,” “my,” or “mine”). The next most used linguistic categories were positive emotion words (mean 4.85%, SD 1.70%) and insight words (mean 4.05%, SD 1.32%). The mean percentage of negations among all words of a user during the whole counseling process was 3.76% (SD 1.54%). Furthermore, negative emotion words were used with a mean percentage among all words of 3.23% (SD 1.27%). Causation words were used with a mean percentage among all words of 2.93% (SD 1.04%). Finally, first-person plural pronouns were least frequently used with a mean percentage among all words of 0.43% (SD 0.63%).

### Gender Differences in Linguistic Variables

Gender-specific descriptive statistics are displayed in Table 2. A 1-way MANOVA showed statistically significant differences in linguistic variables between genders (F_14, 11,932_=8.945; *P*<.001; partial η²=0.01; Wilk Λ=0.979). Post-hoc univariate ANOVAs were conducted separately for every linguistic variable. Separate ANOVAs and respective Bonferroni-corrected post-hoc tests showed that when controlling for word count, there were statistically significant differences in the use of first-person singular pronouns between genders (F_2, 5972_=49.780; *P*<.001; partial η²=0.02), with female users (mean difference=0.90, 95% CI 0.68-1.12; *P*<.001) and users identifying as diverse (mean difference=0.98, 95% CI 0.37-1.56; *P*<.001) using more first-person singular pronouns than male users, whereas there was no significant difference between female users and users identifying as diverse (mean difference=0.08, 95% CI −0.66 to 0.50; *P*>.99). Furthermore, the use of negations differed significantly between genders (F_2, 5972_=4.915; *P*=.007; partial η²=0.002), with female users using significantly more negations than male users (mean difference=−0.16, 95% CI 0.03-0.30; *P*=.01), while there were no significant differences in the use of negations between male users and users identifying as diverse (mean difference=0.329, 95% CI −0.05 to 0.71; *P*=.12) and between female users and users identifying as diverse (mean difference=0.17, 95% CI −0.20 to 0.53; *P*=.84). Another significant difference was found in the use of negative emotion words between genders (F_2, 5972_=4.505; *P*=.01; partial η²=0.00), with female users using significantly more negative emotion words than male users (mean difference=0.12, 95% CI 0.01-0.23; *P*=.04; not significant after Bonferroni correction), while no significant differences in the use of negative emotion words were found between female users and users identifying as diverse (mean difference=0.22, 95% CI −0.07 to 0.52; *P*=.22) and between male users and users identifying as diverse (mean difference=0.11, 95% CI −0.42 to 0.21; *P*>.99). Finally, the results showed a significant difference in the use of insight words between genders (F_2, 5972_=15.215; *P*<.001; partial η²=0.01), with female users (mean difference=0.26, 95% CI 0.14-0.37; *P*<.001) and users identifying as diverse (mean difference=0.41, 95% CI −0.15 to 0.47; *P*=.007) using significantly more insight words than male users, while no significant differences were found between female users and users identifying as diverse (mean difference=0.16, 95% CI −0.42 to 0.21; *P*=.67). No overall significant differences were found between genders in the use of first-person plural pronouns (F_2, 5972_=3.006; *P*=.05; partial η²=0.00), positive emotion words (F_2, 5972_=0.489; *P*=.61; partial η²=0.00), and causation words (F_2, 5972_=2.434; *P*=.09; partial η²=0.00).

**Table 2:** Gender-specific differences in language usage (N=5978).

Linguistic variables	Male^a^ (n=881), mean (SD)	Female^a^ (n=4988), mean (SD)	Diverse^a^ (n=109), mean (SD)	*P* value
First-person singular pronouns	10.81 (2.60)^b^	11.71 (2.43)^c^	11.79 (2.55)^c^	<.001
First-person plural pronouns	0.47 (0.84)	0.42 (0.59)	0.32 (0.43)	.05
Negations	3.62 (2.24)^b^	3.76 (1.39)^c,d^	3.94 (1.49)^b,d^	.007
Positive emotions	4.87 (1.80)	4.86 (1.67)	4.68 (1.79)	.61
Negative emotions	3.13 (1.37)^b^	3.24 (1.24)^c^	3.02 (1.43)^b,c^	.01
Insight words	3.81 (1.38)^b^	4.07 (1.30)^c^	4.20 (1.42)^c^	<.001
Causation words	2.99 (1.12)	2.92 (1.02)	3.04 (0.92)	.09

^a^Reduced sample size owing to missing data on gender.

^b,c,d^Different letters indicate significant differences between the groups, while same letters indicate no significant differences between the groups*.*

### Predicting Psychiatric Symptoms by Linguistic Variables

The binomial logistic regression model was statistically significant (χ²_8_=25.0; *P*=.002), resulting in a small amount of explained variance, as shown by Nagelkerke R^2^=0.124 (Table 3). Of the 10 variables entered into the regression model, all but 3 contributed significantly to the presence of psychiatric symptoms: first-person singular and plural pronouns, negations, negative emotion words, causation words (all *P*<.001), and female gender (*P*=.005), while positive emotion words (*P*=.08), insight words (*P*=.90), and diverse gender (*P*=.57) showed no significant effects. Using first-person plural pronouns was associated with a lower likelihood of reporting psychiatric symptoms (odds ratio [OR] 0.39), as did using more causation words (OR 0.90). In contrast, a higher use of first-person singular pronouns was associated with an increased likelihood of reporting psychiatric symptoms (OR 1.05), as did using more negations (OR 1.11) or negative emotion words (OR 1.15). Finally, being female (OR 1.18) or having a higher age (OR 1.04) was also associated with an increased likelihood of the presence of psychiatric symptoms.

**Table 3:** Prediction of psychiatric symptoms by language usage (N=6962).

Variable			*B* ^a^		SE		Wald^b^		*P* value		OR^c^ (95% CI)
Age			0.04		0.01		22.04		<.001		1.04 (1.02-1.06)
**Gender**											
	Female	0.16		0.06		7.94		.005		1.18 (1.05-1.32)	
	Diverse	0.12		0.21		0.33		.57		1.13 (0.75-1.70)	
First-person singular pronouns			0.05		0.01		15.78		<.001		1.05 (1.03-1.08)
First-person plural pronouns			−0.94		0.05		301.21		<.001		0.39 (0.35-0.44)
Negations			0.11		0.02		32.14		<.001		1.11 (1.07-1.16)
Positive emotions			−0.03		0.02		3.09		.08		0.97 (0.95-1.01)
Negative emotions			0.14		0.02		43.45		<.001		1.15 (1.11-1.21)
Insight words			−0.01		0.02		0.02		.90		1.00 (0.96-1.04)
Causation words			−0.11		0.03		13.80		<.001		0.90 (0.85-0.94)
Constant			−0.87		0.24		13.07		<.001		0.42 (N/A^d^)

^a^*B*: regression coefficient.

^b^Degrees of freedom were 1 for all Wald statistics.

^c^OR: odds ratio.

^d^N/A: not applicable.

The results of an additional correlation analysis between all linguistic variables are reported in Table 4. Among others, significant findings included a negative association between first-person singular pronouns and first-person plural pronouns (*ρ*=−0.24; *P*<.001). In line with this, first-person singular pronouns were positively correlated with negations (*ρ*=0.17; *P*<.001) and negative emotion words (*ρ*=0.15; *P*<.001).

**Table 4:** Spearman correlation coefficients between linguistic variables (N=6962).

Variable			First-person singular pronouns		First-person plural pronouns		Negations		Positive emotions		Negative emotions		Insight words		Causation words
**First-person singular pronouns**															
	*ρ*	1		−0.24^a^		0.17^a^		0.04^a^		0.15^a^		0.29^a^		−0.02	
	*P* value	—^b^		<.001		<.001		.002		<.001		<.001		.22	
**First-person plural pronouns**															
	*ρ*	−0.24^a^		1		−0.08^a^		−0.01		−0.10^a^		−0.03^a^		−0.07^a^	
	*P* value	<.001		—		<.001		.34		<.001		.01		<.001	
**Negations**															
	*ρ*	0.17^a^		−0.08^a^		1		−0.02^a^		0.02		0.13^a^		0.04^a^	
	*P* value	<.001		<.001		—		.045		.19		<.001		<.001	
**Positive emotions**															
	*ρ*	0.04^a^		−0.01		−0.02^a^		1		−0.06^a^		−0.03^a^		0.04^a^	
	*P* value	.002		.34		.045		—		<.001		.023		<.001	
**Negative emotions**															
	*ρ*	0.15^a^		−0.10^a^		0.02		−0.06^a^		1		0.03^a^		−0.07^a^	
	*P* value	<.001		<.001		.19		<.001		—		.023		<.001	
**Insight words**															
	*ρ*	0.29^a^		−0.03^a^		0.13^a^		−0.03^a^		0.03^a^		1		0.04^a^	
	*P* value	<.001		<.01		<.001		.023		.023		—		<.001	
**Causation words**															
	*ρ*	−0.02		−0.07^a^		0.04^a^		0.04^a^		−0.07^a^		0.04^a^		1	
	*P* value	.22		<.001		<.001		<.001		<.001		<.001		—	

^a^Statistical significance.

^b^Not applicable.

### Associations Between Linguistic Variables and Psychiatric Symptoms

Finally, an exploratory correlation analysis indicated evidence for the relationship between linguistic variables and various psychiatric symptoms, which are displayed in Table 5. Suicidality, self-harm, depression, and anxiety showed the most significant correlations to linguistic variables. In particular, the use of first-person singular pronouns was positively associated with suicidality (*ρ*=0.11; *P*<.001) and self-harm (*ρ*=0.10; *P*<.001). The use of first-person plural pronouns was negatively associated with suicidality (*ρ*=−0.10; *P*<.001) and depression (*ρ*=−0.14; *P*<.001). The use of negations was positively associated with suicidality (*ρ*=0.18; *P*<.001) and self-harm (*ρ*=0.12; *P*<.001). Finally, the use of negative emotion words was positively associated with depression (*ρ*=0.12; *P*<.001) and anxiety (*ρ*=0.15; *P*<.001).

**Table 5:** Spearman correlation coefficients between linguistic variables and psychiatric symptoms (N=6962).

Variable		Suicidality	Self-harm	Depression	Anxiety	Eating disorder symptoms	Flashbacks	Obsessive-compulsive symptoms	Addictive behavior
**First-person singular pronouns**									
	*ρ*	0.11^a^	0.10^a^	0.08^a^	−0.01	0.05^a^	−0.02	0.00	−0.01
	*P* value	<.001	<.001	<.001	.46	<.001	.08	.75	.33
**First-person plural pronouns**									
	*ρ*	−0.10^a^	−0.08^a^	−0.14^a^	−0.04^a^	−0.04^a^	−0.01	−0.03^a^	−0.04^a^
	*P* value	<.001	<.001	<.001	<.001	<.001	.26	.007	<.001
**Negations**									
	*ρ*	0.18^a^	0.12^a^	−0.06^a^	−0.06^a^	0.01	−0.02	−0.01	0.00
	*P* value	<.001	<.001	<.001	<.001	.40	.07	.28	.80
**Positive emotions**									
	*ρ*	−0.05^a^	−0.02	0.01	0.00	−0.02	−0.02	−0.02	−0.03^a^
	*P* value	<.001	.08	.41	.87	.06	.19	.09	.01
**Negative emotions**									
	*ρ*	0.04^a^	0.03^a^	0.12^a^	0.15^a^	−0.03^a^	0.02	0.02^a^	0.00
	*P* value	.002	.03	<.001	<.001	.02	.18	.045	.82
**Insight words**									
	*ρ*	0.03^a^	−0.00	0.08^a^	−0.01	−0.01	−0.01	0.00^a^	−0.04^a^
	*P* value	.005	.73	<.001	.65	.67	.61	.045	<.001
**Causation words**									
	*ρ*	−0.06^a^	−0.03^a^	−0.03^a^	−0.02	−0.02	−0.04^a^	0.01	−0.01
	*P* value	<.001	.007	.03	.10	.13	<.001	.63	.32

^a^Statistical significance.

## Discussion

### Principal Findings and Comparison With Prior Work

The findings of this study provide first-time valuable insights into the psycholinguistic characteristics of children, adolescents, and young adults seeking psychosocial support through a messenger-based crisis counseling service (*krisenchat*). Previous findings that examined psycholinguistic characteristics in association with mental health, which however focused on texts (eg, from social media or online therapies), could be identified in the chat context as well. Specifically, linguistic variables were found to be associated with the presence of psychiatric symptoms [43,44,57]. The use of first-person singular pronouns, negations, and negative emotion words increased the likelihood of the presence of psychiatric symptoms, while the use of first-person plural pronouns and causation words was associated with a lower likelihood of the presence of psychiatric symptoms. Female gender was also associated with an increased likelihood of the presence of psychiatric symptoms, which is consistent with the higher prevalence of psychiatric symptoms in women [58]. Gender differences were found, with female users exhibiting more frequent use of certain linguistic features. Previous linguistic analyses, especially in the digital context, such as social media platforms, have been performed with a focus on the presence of psychiatric symptoms or associations with psychiatric symptoms, in particular, depressive symptoms [59]. Linguistic analyses of social media have proven useful in predicting depression, anxiety, loneliness, personality disorders, or other mental health issues [43,57,60-62]. As there are no other comparative studies in this field, the focus of the below comparison of the present results with previous findings relies on correlates of linguistic variables with the presence of psychiatric symptoms, especially depression or anxiety.

#### First-Person Pronouns

Starting with the most frequently used linguistic variable among those examined, an increased use of first-person singular pronouns was associated with a higher likelihood of the presence of psychiatric symptoms. Additionally, it was determined that they were used more often by female users and users identifying as diverse than by male users. In contrast, the linguistic variable of first-person plural pronouns was found to be the least used and did not predict the presence of psychiatric symptoms. Taking into account the most frequently mentioned concerns among the users of *krisenchat* (see [20]), involving psychiatric symptoms, such as depression or anxiety, and the higher prevalence of depression in female samples [63], these findings are consistent with previous findings on language usage. Thus, in line with cognitive theories of depression (eg, according to [33]) indicating that depression is associated with an increased self-focus, previous research showed that individuals with depression used more first-person singular pronouns (ie, “I,” “my,” “me,” and “mine”) in both spoken and written language [33,34]. Similarly, first-person plural pronouns (ie, “we,” “us,” and “our”) were used significantly less by depressed individuals, which may be attributed to social isolation or lack of social integration and social engagement in the context of depression [64-66]. However, the increased use of first-person singular pronouns may also be a marker for increased vulnerability to stress and negative emotionality and not directly for depression [67,68].

#### Negations and Causation Words

Negations were found to be significantly associated with the presence of psychiatric symptoms and were used more frequently by female users than male users or users identifying as diverse. Contradicting the hypothesis, the present results indicate that the use of causation words reduces the likelihood of the presence of psychiatric symptoms, while no significant differences were found between genders. In line with this finding, previous studies indicated that low use of causation words is associated with positive treatment outcomes in treatment for personality disorders [53]. It was found that the use of fewer cognitive words, such as causation words, was associated with a more coherent personal story [69]. A recent study underlined this finding by pointing out that patients having depression tend to use significantly more aligned sentences than bringing them into a logical chain compared with a healthy control group [34]. This cross-sectional study design does not allow to draw conclusions about the trend of the use of causation words throughout the chat counseling. The meaning of these divergent results deserves further specific longitudinal research on the development and change of language use across chat counseling.

#### Negative and Positive Emotion Words

Elevated use of negative emotion words was associated with an increase in the presence of psychiatric symptoms. Negative emotion words were found to differ between genders, that is, more negative emotion words were used by female users than by male users. This is in line with previous research indicating a significantly higher general use of emotion words by women than men, while men were found to use more anger words [70,71]. This is also in accordance with findings showing that more frequent use of negative emotion words, including anxiety, sadness, and anger words, was positively correlated with higher anxiety and depression levels [54].

#### Depressive Symptoms

Taken together, the results underline a higher likelihood of depression (or anxiety) in users using more self-focused language (first-person singular pronouns), and more negative and fewer positive (emotion) words. The response style theory of rumination in depression, which could also be proven for worry in generalized anxiety, explains that symptoms of repetitive self-focused negative thinking become habitual over time [72,73]. In terms of language usage, this theory suggests that people with high levels of depression or anxiety might communicate using more self-focused language (ie, more I-related pronouns), and more negative and fewer positive words, and that this tendency may become habitual and outside of conscious awareness. In line with this, linguistic analyses of text-based therapy found reductions in the use of first-person singular pronouns, even though language usage was not being focused on in the treatment [74]. Moreover, changes in the use of positive and negative emotion words and words indicating certainty (eg, “always” and “never”) could be identified during the treatment for depression. Researchers interpret these findings as changes in cognitive processes [75].

#### Suicidality

In relation to depression, it is also important to keep suicidality in mind because messenger-based chat counseling services are used in acute crises, such as suicidality [76]. In accordance with the present findings, previous studies found that suicidal behavior is associated with the use of more I-related pronouns [77]. Likewise, in accordance with the present findings, previous literary analyses indicated that suicidal poets also used fewer first-person plural pronouns than nonsuicidal poets [66]. In addition, these studies showed that the use of more absolutist language, that is, superlatives and intensifiers (eg, “absolutely,” “completely,” “all,” “none,” etc), was associated with suicidality [60,77,78].

The results of this study indicate that by considering language usage, differences in the user population can be discovered and may also be linked to psychopathology. Thus, language usage should be integrated into the counseling strategy. In the context of computer-based analyses, it was found that in addition to the standardized diagnostic tools used to confirm a psychiatric diagnosis, linguistic research showed that systematic analyses of clients’ language may be used to reliably classify them into diagnostic groups [34]. Additionally, computer-based methods were shown to distinguish persons with depression from other clinical subgroups [79]. Thus, linguistic or, in general, qualitative analyses of text messages seem to be advisable to examine chat-based counseling services in more depth. This is even more true than for social media platforms because text messages are less influenced by social desirability, facilitating more granular visibility into changes in linguistic patterns [80].

### Implications for Counselors in Chat-Based Counseling

Multiple implications for psychosocial chat counseling have emerged from the findings. First, counselors may use psycholinguistic analyses as an additional tool for assessing the mental health state of users. By monitoring the usage of specific linguistic features, counselors can identify individuals at risk for psychiatric symptoms and tailor interventions accordingly. For example, in cases of elevated use of first-person singular pronouns, negations, and negative emotion words, particularly for female users and users who identify as diverse, a more in-depth exploration of psychiatric symptoms, especially depression, anxiety, or suicidality, is recommended. Regardless of the actual cause, it seems advisable to monitor the frequency of first-person singular pronouns throughout the chat history. For example, establishing a word-counting function during chat counseling may provide counselors with additional valuable information. In turn, increased use of first-person plural pronouns as well as causation words can be identified as protective factors, and also expanded and used as such. For example, increased use of first-person plural pronouns on the counselor’s side might be helpful to create a sense of “unity” or belongingness. Taking social belongingness into account as a protective factor for depression, suicidality, or mental disorders in general, it seems advisable to create closeness or sociality through language to initially stabilize the user emotionally, for example, meet their need for attachment or create a base to make further recommendations [81].

Furthermore, it can be helpful to mirror statements of users in the context of active listening (positively paraphrased) or to guide the users through targeted questioning techniques into positively formulated thoughts, goals, behavioral directions, etc (ie, in concrete terms) to avoid negations. This implication is also underscored by the fact that increased use of words referring to expectation, trust, and belongingness was associated with lower depression rates [80,81]. Furthermore, the establishment of logical chains, in the sense of causation, also appears to be of great importance. Thus, attempts should be made to bring concerns, feelings, etc into a logical coherent context in the sense of stimulus-reaction chains. Moreover, the use of insight words shows no influence on the presence of psychiatric symptoms, yet reflective functioning is known to be a protective factor [82]. An emphasis on self-reflection could potentially be integrated into psychoeducation by supporting an understanding of one’s own mind as an aspect of resilience and personal agency that can be fostered through social support and professional help [82]. In practice, repeated questions about what feeling is associated with particular problems or concerns, or identifying thoughts or cognition in terms of one’s own belief patterns, can help to practice such skills.

### Strengths and Limitations

To the best of our knowledge, this study is the first to examine language usage in a messenger-based crisis counseling service among youth and young adults. In addition to the strength of the large sample size, this study acts as a reference and comparison for further studies in this area, in part because of the use of the internationally recognized method LIWC. Nevertheless, some limitations have to be taken into account. Owing to the retrospective study design, the data rely on convenience sampling, which limits the generalizability to a more mixed-gender population. It should also be noted that the counseling service is offered in German-speaking countries, which is why cultural and linguistic differences in language usage must be taken into account, and generalizability is limited. An international comparison between counseling services in different countries, cultures, and languages would provide insights into similarities and differences. Assuming that people in crisis reach out to the chat counseling service, high use of emotion words seems somewhat expected. Since the nature of the study was cross-sectional and the words were counted across all chat messages (ie, not in chronological order or within sessions), no data can be provided on the trend of the word count for either positive or negative emotion words, which is why no indications can be derived on whether the word count relates only to the beginning of the chat or also to the progression throughout the chat counseling. Longitudinal studies examining changes in language use across consecutive chat sessions may provide further insights into these associations. Consideration must also be given to the nested data structure, which cannot be clearly read owing to the format used to provide data by LIWC. Therefore, for future studies, in addition to the trend of the word count throughout the chat counseling, the consideration of levels (eg, within a message and during a session) is recommended. Therefore, a qualitative analysis of the chats may be beneficial for providing more in-depth insights into individual language usage as well as concerns. A qualitative approach would also ensure the quality of the classification of the presence of psychiatric symptoms. Likewise, no standardized measurement instruments were used, which in turn opens up further opportunities for future research, for example, implementing symptom-specific questionnaires to examine associations between (changes in) symptom severity and language usage.

### Conclusion

This study underlines the options, possibilities, and chances of examining psycholinguistic characteristics in psychosocial online chat counseling services for children and adolescents. The identified associations between specific linguistic features and the presence of psychiatric symptoms provide valuable insights for the development of targeted interventions. By considering psycholinguistic findings in the counseling practice, counselors may enhance their understanding of the psychological processes of users and their interventions to offer a more targeted service for children and adolescents seeking help. Nevertheless, further research is needed to investigate the mechanisms underlying linguistic patterns and explore the effectiveness of linguistic-based interventions. At the same time, this would allow further research on the link between specific indicators and changes in specific psychopathology.

## Abbreviations

LIWC: Linguistic Inquiry and Word Count
OR: odds ratio
